# The diagnostic accuracy of pharmacological stress echocardiography for the assessment of coronary artery disease: a meta-analysis

**DOI:** 10.1186/1476-7120-6-30

**Published:** 2008-06-19

**Authors:** Eugenio Picano, Sabrina Molinaro, Emilio Pasanisi

**Affiliations:** 1 CNR, Institute of Clinical Physiology, Pisa, Italy

## Abstract

**Background:**

Recent American Heart Association/American College of Cardiology guidelines state that "dobutamine stress echo has substantially higher sensitivity than vasodilator stress echo for detection of coronary artery stenosis" while the European Society of Cardiology guidelines and the European Association of Echocardiography recommendations conclude that "the two tests have very similar applications". Who is right?

**Aim:**

To evaluate the diagnostic accuracy of dobutamine versus dipyridamole stress echocardiography through an evidence-based approach.

**Methods:**

From PubMed search, we identified all papers with coronary angiographic verification and head-to-head comparison of dobutamine stress echo (40 mcg/kg/min ± atropine) versus dipyridamole stress echo performed with state-of-the art protocols (either 0.84 mg/kg in 10' plus atropine, or 0.84 mg/kg in 6' without atropine). A total of 5 papers have been found. Pooled weight meta-analysis was performed.

**Results:**

the 5 analyzed papers recruited 435 patients, 299 with and 136 without angiographically assessed coronary artery disease (quantitatively assessed stenosis > 50%). Dipyridamole and dobutamine showed similar accuracy (87%, 95% confidence intervals, CI, 83–90, vs. 84%, CI, 80–88, p = 0.48), sensitivity (85%, CI 80–89, vs. 86%, CI 78–91, p = 0.81) and specificity (89%, CI 82–94 vs. 86%, CI 75–89, p = 0.15).

**Conclusion:**

When state-of-the art protocols are considered, dipyridamole and dobutamine stress echo have similar accuracy, specificity and – most importantly – sensitivity for detection of CAD. European recommendations concluding that "*dobutamine and vasodilators (at appropriately high doses) are equally potent ischemic stressors for inducing wall motion abnormalities in presence of a critical coronary artery stenosis*" are evidence-based.

## Background

Pharmacological stress echocardiography is widely used for the diagnosis of coronary artery disease [1,2], and the two most employed pharmacological stresses are dipyridamole and dobutamine, first proposed more than 20 years ago [3,4]. The latest 2006 European Society of Cardiology (ESC) guidelines for stable angina conclude that *"the two tests have very similar applications and the choice as to which is employed depends largely on local facilities and expertise*" [5]. This statement was corroborated by a meta-analysis of the published literature, included in the guidelines, and showing comparable accuracy, sensitivity and specificity of dobutamine and vasodilator stress echocardiography. However, and paradoxically, on the basis of the same existing literature, the American Heart Association/American College of Cardiology (AHA/ACC) guidelines stated that "*dobutamine stress echo has higher sensitivity than vasodilator stress echo for detection of coronary artery disease" *[6,7]. The recent 2007 recommendations on stress echocardiography of the American Society of Echocardiography conclude that "*although vasodilators may have advantages for assessment of myocardial perfusion, dobutamine is preferred when the test is based on assessment of regional wall motion*" [8]. Who is right? The question has profound clinical relevance, since tens of millions of cardiac stress testing are performed each year [9], and the projected rises is of + 4,900% in the next decade or so [10]. In addition, pharmacological stress imaging with simultaneous assessment of perfusion and function is also at the basis of the growing application of stress-CMR imaging [11]. A source of ambiguity is represented by the presence of several different protocols of vasodilator stress echo proposed over the years, in the continuing quest of the ideal accuracy: one protocol is suitable for perfusion imaging [12,13], another for viability detection [14], and still another one for ischemia induction [15-17]. When true ischemia and regional wall motion abnormalities are the diagnostic end-point, we need high dipyridamole doses (0.84 mg/kg), either with atropine co-administration [16] or with a fast infusion rate [17]. Any sound meta-analysis should only include these state-of-the-art protocols, present in the literature since 15 years [17], in a head-to head comparison with dobutamine stress echo on consecutive populations studied in the same laboratories and with angiographic verification independent of stress results.

## Methods

### Study selection

We designed our search to identify all studies assessing the comparison between dipyridamole and dobutamine stress echocardiography state of the art protocols in their diagnostic accuracy. We conducted a PubMed search from 1985 through 2007 combining stress echocardiography (2777 citations) AND diagnosis (2665 citations) AND dobutamine (1659 citations) AND dipyridamole (201 citations). In a second step we excluded "prognosis" (143 citations). After limiting to human studies we identified 86 citations. There was no language restriction used. Meta-analysis, editorials, letters have been excluded. We only considered original papers addressing head to head comparison between dobutamine stress echo (40 mcg/kg/min ± atropine) and dipyridamole stress echo with state of the art protocols (0.84 mg/kg plus atropine or 0.84 mg/kg in 6 minutes without atropine). The inclusion criteria for this meta-analysis were:

(1) dipyridamole and dobutamine stress echocardiography were performed on the same population of patients, on different days and in random order;

(2) the 2 tests were performed under identical anti-ischemic therapy, if any;

(3) coronary angiography information was used as a reference standard.

Based on this, 5 articles have been selected (from Serbia, Holland, Spain, Italy and Finland) totalling 435 patients with coronary angiography for evaluation of diagnostic accuracy.

**Figure 1:**
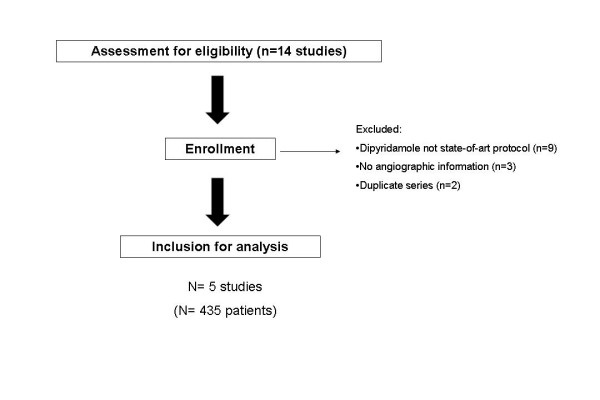
The flow-chart of selection of source studies for the meta-analysis.

We followed the QUORUM guidelines on the reporting of meta-analysis [18]. The selection process of the relevant literature is summarized in Figure 1. Studies performed with protocols not considered today as state-of-art (such as high dose dipyridamole in 10' without atropine) have been excluded [18-26]. Studies without angiographic information and with only prognostic information available were also excluded [27-29]. Studies from the same group were considered only once, to avoid partial re-counting of data [29,30] and only the latest, and largest, study was used as source study. Based on this selection criteria, 5 source studies have been selected (from Serbia, Holland, Spain. Italy and Finland) totalling 435 patients with coronary angiography for evaluation of diagnostic accuracy [31-35]. A vessel was considered to have a significant obstruction ≥ 50% by quantitative or visual analysis (Table. 1). At time of the tests, 282 patients were off anti-ischemic therapy; 77 patients had history of previous myocardial infarction.

### Data extraction

The following data were extracted per source study: author, journal, year of publication, type of test, total number of patients, mean age, proportion of men, proportion of previous myocardial infarction, prevalence of significant disease, definition of significant disease, summary estimates of sensitivity and specificity. In addition to these variables, the absolute number of true-positive, false-negative, false-positive, and true-negative results were extracted per source study.

### Data synthesis

The pooled weighted estimation of sensitivity, specificity and accuracy were reported in Table. 2. Calculations of sensitivity, specificity and accuracy have been performed according to standard definitions and goal of a meta-analysis, with the corresponding 95% confidence intervals (CI). We also calculated the pooled values of sensitivity, specificity and accuracy weighted for sample size with fixed effect model (Comprehensive Meta-Analysis program – Biostat Englewood, NJ). Differences in sensitivity, specificity and accuracy have been compared by the odds-ratio statistics. We expressed continuous data as mean ± SD, and dichotomous variables as percentages. We considered statistically significant a P value < 0.05.

## Results

**Figure 2:**
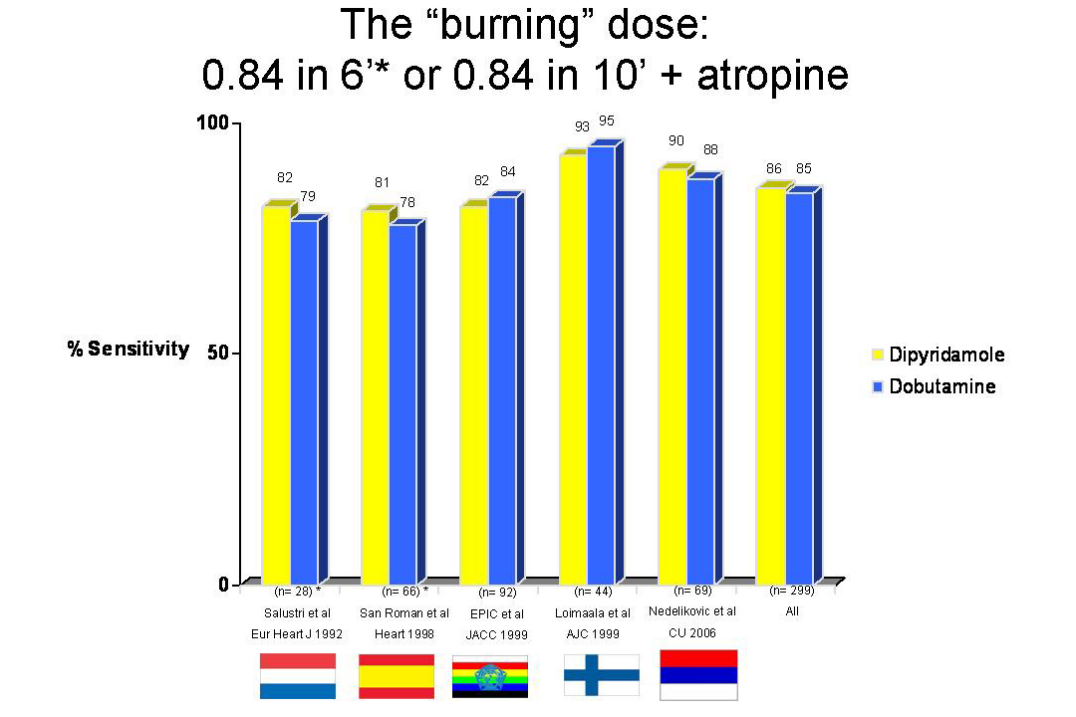
Sensitivity values in individual studies and cumulative analysis.

**Figure 3:**
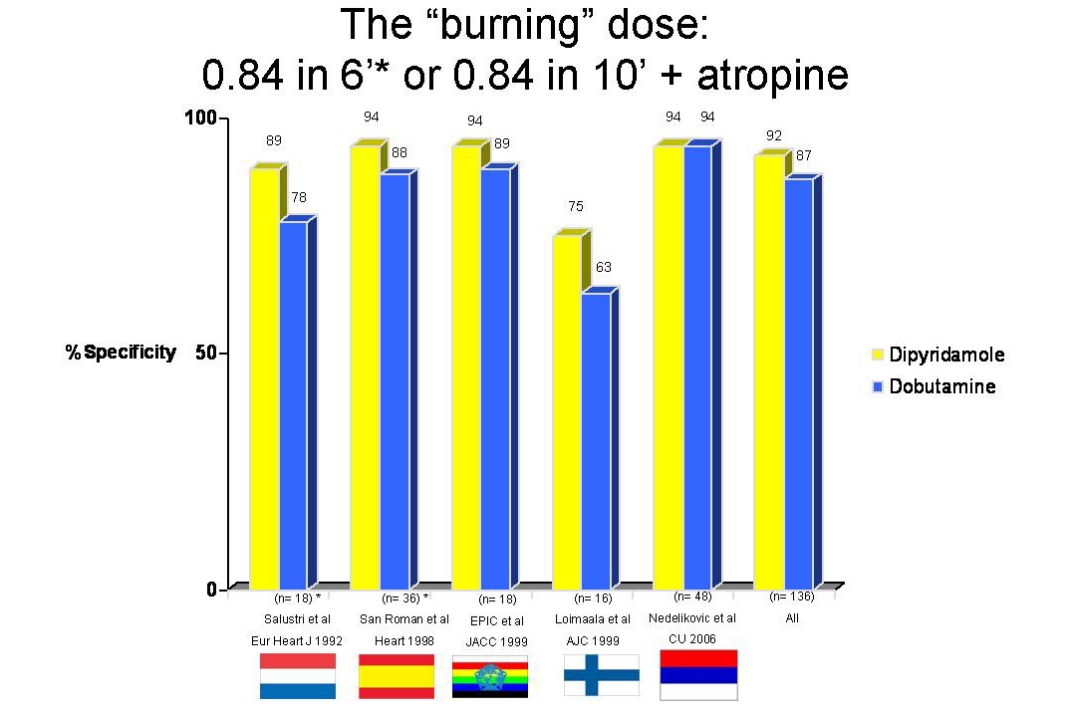
Specificity values in individual studies and cumulative analysis.

**Figure 4:**
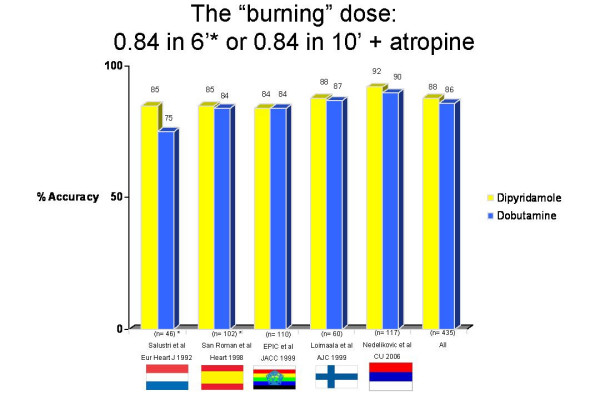
Accuracy values for individual studies and cumulative analysis.

Individual absolute numbers and percent values for each study are reported in Table 1. Standard dose dobutamine protocol (40 mcg/Kg/min) plus atropine was used in all 5 articles. High dose dipyridamole protocol (0.84 mg/kg in 10 minutes) with atropine was employed in 3 studies and 287 patients, and the fast accelerated protocol (0.84 mg/kg in 6 minutes) in 2 studies and 148 patients. Raw data of sensitivity (Figure 2), specificity (Figure 3), and accuracy (Figure 4) values for individual articles and in overall cumulative analysis were not significantly different. Variance-weighted pooled analysis is shown in Table 3, again showing similar values between the two tests.

## Discussion

When state-of-the art protocols are considered, dipyridamole and dobutamine stress echo have similar accuracy, and – most importantly – the same sensitivity for detection of CAD.

### Comparison with previous studies

Several previous meta-analysis pooled data of dipyridamole stress echocardiography, including standard dose with high dose and high dose plus atropine [36-39]. All these studies concluded that dipyridamole has a higher specificity than dobutamine, with a trend to lower sensitivity in less severe forms of single vessel disease. In the most recent and updated of these meta-analyses, Heijenbrook et al. analyzed 58 patients series with dipyridamole echo and 102 with dobutamine echo, and showed a very similar overall accuracy, with higher specificity for dipyridamole (94.6% vs. 84.1% of dobutamine) and higher sensitivity for dobutamine (81% for dobutamine and 71.9% for dipyridamole) [39]. These data can be easily reconciled with the findings of the present study, since the inclusion of old, now obsolete, vasodilator protocols, such as low dose, or high dose without atropine, decreases sensitivity without affecting specificity [26]. Our data also explain the recent findings of stress-CMR, conceptually germane but less operator-dependent than stress echo, showing that the fast high dose dipyridamole protocol is the best choice to catch "two birds with a stone", i.e. to image function and perfusion ("two birds") in one sitting with a single stress ("one stone") [40,41]. This approach is obviously simpler than the "two birds, two stones" approach (with separate testing of perfusion with adenosine and function with dobutamine). It is however imperative that your "stone" (stress) is of sufficient weight (high cumulative dose) and thrown with sufficient speed (fast infusion rate) in order to catch the two birds.

### Pathophysiological basis

**Figure 5:**
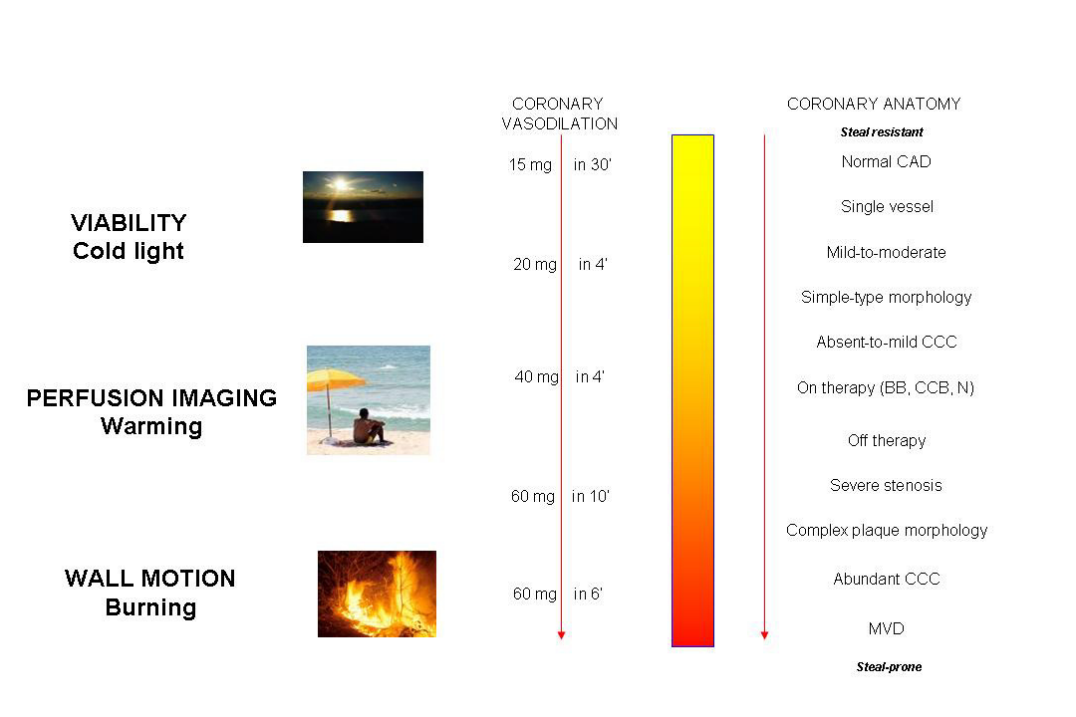
The pathophysiological effects of dipyridamole at different dose windows and as a function of the underlying coronary anatomy in the individual patient. The pro-ischemic, myocardial "burning" effects dominates at the higher doses; the cardioprotective, "cold light" effect at very low doses, and the "warming" hyperemic effect at intermediate doses.

It seems counterintuitive that dipyridamole is a strong coronary vasodilator which does not importantly increase myocardial oxygen demand and is also a powerful ischemic stressor. It is conventional wisdom that "*as long as the oxygen demand is not increased in these segments there is no ischemia and consequently no wall motion abnormality*" [42]. It can appear even more puzzling that the same active principle, given intravenously, is an effective anti-ischemic drug [43], a viability test capable to recruit contractile reserve through a direct metabolic cardioprotective effect [14], a hyperaemic stressor with limited capacity to evoke ischemia [12,13] and – at high, fast doses – a strong ischemic stress [44]. Dipyridamole looks like a character of an Agatha Christie's novel."*Perfectly*", said Poirot, "*The matter begins to clear up wonderfully! The murderer was a man of great strength – he was feeble – it was a woman – it was a right-handed person – he was a left-handed person. Ah, C'est rigolo, tout ça!*" [45]. The results of this meta-analysis may help us to enter the second half of the Agatha Christie's novel. Dipyridamole acts through accumulation of endogenous adenosine, which is a key retaliatory metabolite [46] with a variety of anti-ischemic and cardioprotective effects [47-49] – but too much of a good thing can be dangerous [50]. A low level, gradual exposure to adenosine – or even a high level exposure in absence of steal prone anatomy – can have exert a powerful anti-ischemic and cardioprotective effect, due to the "cold light" of direct cardioprotective effects independent of flow increase, mainly mediated by stimulation of high affinity A1 and A3 myocardial receptors [50]. A higher level of adenosine accumulation will induce a stronger hyperemic effect: warm coronary vasodilation that will produce a differential tanning (myocardial tracer uptake or coronary flow increase) of regions perfused by coronary stenoses of different severity. This effect is achieved with standard doses, through stimulation of A2a adenosine receptors on coronary arterioles smooth muscle cells and is convenient for hyperemic imaging [50]. At high doses, the A2a-mediated pro-ischemic effect prevails. The exposure of steal-vulnerable myocardium to excessive amount of adenosine will "burn" the myocardium, with ischemia induction which is the necessary end-point for vasodilator stress echocardiography (Figure 5). In this context, the vulnerable "phototype" is represented by the presence of a coronary anatomy with tight stenosis (necessary for vertical steal phenomena), especially with complex-type morphology (with endothelial damage reducing the epicardial dilatory effects of adenosine), and abundant coronary collateral circulation (i.e., the anatomical background enhancing horizontal-steal phenomena). The burning effects of excessive coronary arteriolar vasodilation can be effectively prevented, or attenuated, by anti-ischemic therapy with beta-blockers, nitrates, or calcium-antagonists, which exert a powerful anti-steal effect, with enhanced redistribution of hyperaemic flow towards the subendocardium [51], acting therefore as a protective "sunshade umbrella" for the steal-prone myocardium. With this conceptual framework, we can enter the second half of the Agatha Christie's novel: the same drug, in the same patient, can have different effects according to the dose employed, the infusion rate, and the underlying coronary anatomy (Fig. 5).

### Study limitations

We did not exclude studies based on the quality of data reported. Juni et al showed that studies should not be excluded based on composite quality scores because many quality scales are more closely related to reporting quality than to the internal validity of the studies [52]. Instead, relevant methodological aspects should be assessed individually and their influence on effect sizes explored. Therefore, we only included studies that used the same anatomic reference standards, i.e. a (visually or quantitatively assessed) coronary artery stenosis ≥ 50% and with remarkably similar methodology regarding the visual assessment of regional wall motion analysis.

Another potential confounder is the publication bias. Stern and Simes have shown that positive results are not only more likely to be published than negative results, but they also have a significant shorter time to publication [53]. However, in this particular case it is not clear what is a positive finding, since 2 stress tests were compared, and the results appear consistent across the different studies, without detectable changes related to the year of the study, the male predominance, the percentage of previous myocardial infarction, the prevalence of CAD, or the concomitance of antianginal therapy. All these factors are known to affect the stress test accuracy in absolute terms – but in this study they are averaged out, since only inter-test differences applied to the same population are considered.

We focused only on diagnostic accuracy. Other aspects of the test are at least equally important and include the prognostic value, the safety, the feasibility rate, the quality of echocardiographic imaging and the capability to recognize myocardial viability. There are extensive data in the literature that the prognostic accuracy of the 2 tests is very similar [28,29,54,55], whereas the number of minor, but limiting, and major life-threatening complications is about 2 times higher with dobutamine than with dipyridamole [56-58]. Submaximal studies are found in 5% patients with dipyridamole, and 10% with dobutamine. Life-threatening complications occur in 1 out of 600 patients with high dose dipyridamole and 1 in 300 with dobutamine [49,50]. Regarding image quality degradation during stress, only 2 studies – both not included in the present metaanalysis since the high dipyridamole dose without atropine was used – addressed semi-quantitatively [23] or qualitatively [21] the issue of image degradation during stress. Sochowsky et al described that a worsening of image quality occurred significantly more frequently during dobutamine than with dipyridamole stress [23], due to tachycardia and hyperventilation. Beleslin et al compared head to head 136 patients with treadmill exercise, dobutamine and dipyridamole stress echo and concluded that "from the technical viewpoint, dipyridamole represents the primary school, dobutamine the secondary school, and exercise the University in the stress echo cursus studiorum" [21]. For recognition of myocardial viability, both tests have similar diagnostic and prognostic value [59,60], but the wealth of data clearly favors low dose dobutamine, which is currently the only stress echo test with this class 1 indication for viability assessment in guidelines [5,6].

## Conclusion

The recent ESC [5] guidelines on stable angina and EAE recommendations on stress echocardiography [61] are evidence-based in concluding that "dobutamine and vasodilators (at appropriately high doses) are equally potent ischemic stressors for inducing wall abnormalities in presence of a critical coronary artery stenosis." The implications are far-reaching for the better understanding of pathophysiology of ischemic heart disease and the practice of cardiac stress testing.
